# Genome-Wide Assessment of Runs of Homozygosity by Whole-Genome Sequencing in Diverse Horse Breeds Worldwide

**DOI:** 10.3390/genes14061211

**Published:** 2023-06-01

**Authors:** Chujie Chen, Bo Zhu, Xiangwei Tang, Bin Chen, Mei Liu, Ning Gao, Sheng Li, Jingjing Gu

**Affiliations:** 1 Hunan Provincial Key Laboratory for Genetic Improvement of Domestic Animal, College of Animal Science and Technology, Hunan Agricultural University, Changsha 410128, China; chencj1995@163.com (C.C.); txw@stu.hunau.edu.cn (X.T.); chenbin7586@hunau.edu.cn (B.C.); mei.liu@hunau.edu.cn (M.L.); gaon@hunau.edu.cn (N.G.); 2 Novogene Bioinformatics Institute, Beijing 100015, China; zhubo@novogene.com; 3 Maxun Biotechnology Institute, Changsha 410024, China

**Keywords:** ROH, whole-genome sequencing, inbreeding, horse, Thoroughbred

## Abstract

In the genomes of diploid organisms, runs of homozygosity (ROH), consecutive segments of homozygosity, are extended. ROH can be applied to evaluate the inbreeding situation of individuals without pedigree data and to detect selective signatures via ROH islands. We sequenced and analyzed data derived from the whole-genome sequencing of 97 horses, investigated the distribution of genome-wide ROH patterns, and calculated ROH-based inbreeding coefficients for 16 representative horse varieties from around the world. Our findings indicated that both ancient and recent inbreeding occurrences had varying degrees of impact on various horse breeds. However, recent inbreeding events were uncommon, particularly among indigenous horse breeds. Consequently, the ROH-based genomic inbreeding coefficient could aid in monitoring the level of inbreeding. Using the Thoroughbred population as a case study, we discovered 24 ROH islands containing 72 candidate genes associated with artificial selection traits. We found that the candidate genes in Thoroughbreds were involved in neurotransmission (*CHRNA6*, *PRKN*, and *GRM1*), muscle development (*ADAMTS15* and *QKI*), positive regulation of heart rate and heart contraction (*HEY2* and *TRDN*), regulation of insulin secretion (*CACNA1S*, *KCNMB2*, and *KCNMB3*), and spermatogenesis (*JAM3*, *PACRG*, and *SPATA6L*). Our findings provide insight into horse breed characteristics and future breeding strategies.

## 1. Introduction

Domestication of horses began approximately 5500 years ago in the Eurasian steppe [1,2,3]. Since then, selective breeding and acclimatization have shaped the horse genome, resulting in more than 500 horse breeds worldwide [4]. Horses are employed in transportation, warfare, agriculture, and entertainment and can be categorized according to their usage (racing, sport, endurance, local, and gait), appearance (body size, coat color, and conformation), and temperament (hot, warm, and cold). Horse genomics has progressed rapidly since the establishment of the horse reference genome [5,6] and advancements in genomics technology. The genetic mechanisms of many horse traits have been investigated using single nucleotide polymorphism (SNP) chips and resequencing of the whole genome [7]. In contrast to SNP chips, whole-genome sequencing can repeatedly cover the entire genome, resulting in greater resolution and accuracy.

Inbreeding is inevitable in the horse population, and breeds subjected to intense artificial selection and/or those with a small population size are more likely to experience the negative effects of inbreeding (such as inbreeding depression). Calculating the inbreeding coefficient from pedigree-based data [8] is the conventional method for measuring the inbreeding level. However, pedigree mistakes in farm animals [9] and horse populations [10] are prevalent. Runs of homozygosity (ROH) are continuous stretches of homozygosity regions spread across diploid genomes resulting from the transmission of identical haplotypes from common ancestors [11]. ROHs were first identified in the human genome [12] and have been used to define the degree of inbreeding [13]. The ROH-based genomic inbreeding coefficient (*F*_ROH_) is described by measuring the proportion of the ratio of the sum of each individual’s ROH lengths to the total genome length [14].

Due to the fact that inbreeding is one of the primary causes of ROH [15], ROH is able to be applied to evaluate the inbreeding situation of individuals without pedigree data. In general, long ROHs indicate recent genome-wide inbreeding events, whereas short ROHs indicate ancient inbreeding. Additionally, population bottlenecks, genetic drift, and selection may contribute to the emergence of ROHs [16]. ROH are not distributed indistinguishably across the genome and accumulate in particular regions of the genome in various populations. The regions of the genome with the highest ROH occurrence in a population are known as “ROH islands” [17]. Genomic regions with selective signatures frequently overlap with ROH islands [18]. ROH islands can therefore be used to identify potentially selected genomic regions and identify the genetic basis of commercially valuable traits in farm animal populations [19]. In recent years, ROH detections on horses have become increasingly prevalent. However, most ROH studies on horses have focused on SNP chip data, and only a few have utilized whole-genome sequencing for ROH analysis [20].

We sequenced and utilized whole-genome sequencing data from 97 horses to identify and analyze ROH patterns in 16 globally representative horse breeds. Using the Thoroughbred population as a case study, we further investigated ROH islands containing potential candidate genes for performance traits. Our findings provide insight into horse breed characteristics and future breeding strategies.

## 2. Materials and Methods

### 2.1. Ethics Statement

The Hunan Agricultural University’s Biomedical Research Ethics Committee approved this study (No. 202046). No horses were injured during or after the sample collection, and they remained healthy.

### 2.2. Sampling and Whole-Genome Sequencing

In our horse panel, 37 horses were whole-genome sequenced at high coverage (~30×). Using a standard phenol-chloroform method, DNAs were obtained from freshly collected blood samples. Following instructions provided by the manufacturer, sequencing libraries were constructed and sequenced using an Illumina HiSeq 4000 sequencer to generate 150 bp paired-end reads. We also retrieved whole-genome sequencing data for more diverse horse breeds from NCBI (BioProject accession numbers: PRJEB10098, PRJEB10854, PRJNA168142, PRJNA205517, PRJNA230019, PRJNA233529, PRJNA288817, and PRJNA291776). We analyzed a diverse horse panel (breed *n* = 16; total sample *n* = 97) with distinct appearances, breed-defining traits, and geographic origins. The horse breeds included Arabian, Andalusian, Akhal-Teke, Criollo, Debao, Friesian, Hanoverian, Jeju, Mongolian, Franches-Montagnes, Przewalskii, American Quarter Horse, Shetland pony, Standardbred, Thoroughbred, and Yakutian. 

### 2.3. Quality Controls and SNP Genotyping

All raw sequencing reads were preprocessed for quality control and filtered using FastQC. After quality control, the BWA program [21] was employed to map clean reads to the equine reference genome (EquCab3). Population-scale SNP calling was performed using the Bayesian approach in the SAMtools package [22]. The EquCab3 genome was used to conduct SNP annotation using ANNOVAR [23]. According to their genomic location, SNPs were classified into the following classes: exonic, intronic, splicing sites, upstream, downstream, and intergenic. Exonic SNPs were further classified as synonymous, non-synonymous, stop-gain, and stop-loss SNPs. 

### 2.4. Runs of Homozygosity Detection

ROH were calculated utilizing Plink v1.9 [24]. We scanned the entire genome of each horse using a sliding window strategy to identify the ROH regions. The criteria used to identify ROH were as follows: (1) the size of the sliding window was set to 500 kb; (2) the lowest SNP density was one per 50 kb; (3) 1 Mb was the maximum distance between SNPs; (4) based on the ROH length, 1 heterozygote was allowed in a sliding window; (5) a maximum of 4 missing genotypes were allowed. The defined ROHs were categorized according to their length: <1 Mb, 1–5 Mb, 5–10 Mb, and >10 Mb. 

### 2.5. Inbreeding Coefficients

As reported by McQuillan et al. [14], genome-wide inbreeding coefficients were computed. In each individual, to calculate the inbreeding coefficients for each of the five ROH categories, the total length of each ROH category was divided by the total length of the autosomes (2280.94 Mb) in the sequenced horse genome. The inbreeding coefficients were recorded as *F*_ROH < 1 Mb_ (<1 Mb), *F*_ROH 1–5 Mb_ (1 to 5 Mb), *F*_ROH 5–10 Mb_ (5 to 10 Mb), *F*_ROH > 10 Mb_ (>10 Mb), and *F*_ROH all_ (including ROHs of all lengths).

### 2.6. Detection of ROH Islands in Thoroughbreds and Candidate Genes

To determine the ROH islands in the Thoroughbred population (*n* = 22), we calculated the frequency of each SNP across all ROH regions in the entire Thoroughbred population. Potential ROH islands were identified as the top 1% of SNPs based on their occurrence frequency in the empirical distribution [17]. Using information from the Ensembl Genome Browser (www.ensembl.org, accessed on 20 February 2023), genes contained in the ROH islands were annotated. Functional analysis of the candidate genes was performed using Gene Ontology (GO) Biological Process enrichment and Kyoto Encyclopedia of Genes and Genomes (KEGG) pathway analyses in DAVID 2021 [25], with an adjusted *p*-value greater than 0.05 indicating significance. 

## 3. Results

### 3.1. Whole-Genome Sequencing 

Using the whole-genome sequencing method, we sequenced and obtained a total of 56,768.2 million clean reads for 97 horse individuals, and the mean entire genome coverage for each horse was 25.6× (Table S1). We obtained 22,539,736 informative SNPs that were evenly dispersed across the equine genome (10 SNPs per kb on average) following a stringent quality control filtering process. Using the Ensembl horse gene annotation set (Release 106), these population SNPs were annotated. A total of 8,461,302 (37.5%) SNPs were mapped within the gene regions, including 7,835,178 SNPs in introns, 208,369 SNPs in exons, and 417,052 SNPs in untranslated regions.

### 3.2. ROHs in the 16 Horse Breeds

**Figure 1:**
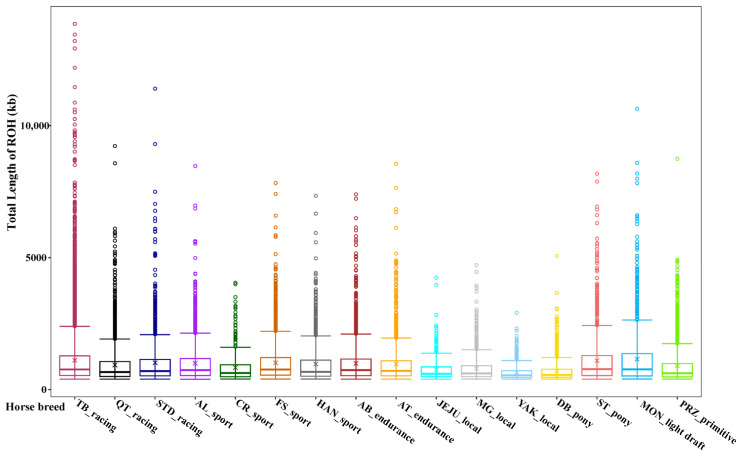
Box plots of ROHs detected in 16 different horse breeds. The horse breeds were classified according to their main usages. The horse breeds included Arabian (AB), Andalusian (AL), Akhal-Teke (AT), Criollo (CR), Debao (DB), Friesian (FS), Hanoverian (HAN), Jeju (JEJU), Mongolian (MG), Franches-Montagnes (MON), Przewalskii (PRZ), American Quarter Horse (QT), Shetland pony (ST), Standardbred (STD), Thoroughbred (TB) and Yakutian (YAK). Hollow dots represent the outliers.

In this study, ROHs were identified in 16 diverse horse breeds that represented different phenotypes and levels subject to selection (Figure 1). To understand the ROH characteristics of the studied horse population, we first examined the average total length of the population ROH and the average number of total ROH for each horse breed. We found that the three highest average numbers of total ROH per horse breed were discovered in three sport horse breeds: Friesian (637), Arabian (621), and Thoroughbred (568). The three lowest average numbers of total ROH per horse were observed in Przewalskii primitive horses (180) and two local horse breeds, Debao (167) and Yakutian (102). 

Furthermore, the average total length of ROH maintained the same pattern as the average number of total ROH for each breed. Friesian horses had the largest average total length of ROH (635.69 Mb), followed by Arabian (602.63 Mb) and Thoroughbred (614.86 Mb). The lowest average total length of ROH was still found in the primitive and local horse breeds (Przewalskii: 159.15 Mb, Debao: 118.61 Mb, and Yakutian: 65.69 Mb).

Of the ROH segments in the four length categories, most are short ROH segments (<1 Mb), followed by ROH segments of 1–5 Mb, accounting for 69.55% and 29.83% of the total number of ROHs, respectively. ROH segments (5–10 Mb) were present in 12 horse breeds, with Thoroughbred having the most abundant (117). ROHs greater than 10 Mb were also the highest in Thoroughbred (10), followed by Standardbreds and Franches-Montagnes, each with only one long ROH. No long ROH fragments (>10 Mb) were found in the other horse breeds. Table 1 provides a summary of the ROH segment statistics for the 16 horse breeds.

### 3.3. Assessment of Inbreeding Coefficients

According to the different ROH length categories, the inbreeding coefficient was calculated for each horse, and then the average inbreeding coefficient within the horse breed was calculated. Friesian had the highest value of *F*_ROH all_ (2.79 × 10^−1^), followed by Arabian (2.64 × 10^−1^) and Thoroughbred (2.58 × 10^−1^). Primitive and indigenous breeds, such as Przewalskii (6.98 × 10^−2^), Mongolian (6.89 × 10^−2^), Debao (5.20 × 10^−2^) and Yakutian (2.88 × 10^−2^), had relatively low inbreeding coefficient values. In the <1 Mb and 1–5 Mb ROH range divisions, Friesian had the highest *F*_ROH (<1 Mb)_ (1.16 × 10^−1^) and *F*_ROH (1–5 Mb)_ (1.59 × 10^−1^), whereas Yakutian had the lowest *F*_ROH (<1 Mb)_ (2.26 × 10^−2^) and *F*_ROH (1–5 Mb)_ (6.20 × 10^−3^) among all the horse breeds. In the 5–10 Mb and >10 Mb long ROH range divisions, Thoroughbreds had the highest inbreeding coefficients *F*_ROH (5–10 Mb)_ (1.42 × 10^−2^) and *F*_ROH (>10 Mb)_ (2.27 × 10^−3^) compared to the rest of the horse breeds. The mean genomic inbreeding coefficients (*F*_ROH_) for ROH of different length categories in horse populations are shown in Table 2.

### 3.4. The ROH Islands and Candidate Genes in Thoroughbreds

**Figure 2:**
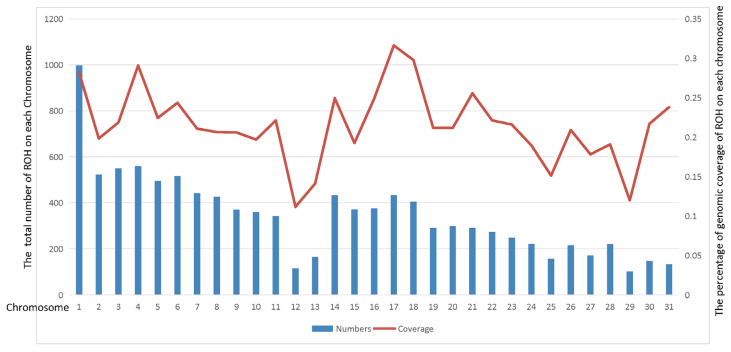
Distribution of ROH in Thoroughbred population. The bars represent the sum of number of ROH, and the line represents the percentage of genomic ROH coverage on horse chromosomes 1 to 31.

Since Thoroughbreds have been selectively bred for racing performance for more than 300 years, we further analyzed the ROH genome-wide distribution patterns using the Thoroughbred population as a case study. In total, 10,631 ROHs were identified in 22 Thoroughbred horses (Table S2). We found that ROH segments were not evenly distributed across chromosomes. Figure 2 displays the number of ROH and percentage of genomic ROH coverage in the Thoroughbred population on each chromosome. With a high coverage ratio of 28.2%, chromosome 1 of Equus caballus (ECA1) contains the most ROH segments (997). In contrast, ECA29 had the fewest ROH segments (102), and its coverage ratio is the second lowest (11.97%). ECA17 had the highest percentage of coverage (31.63%), while ECA12 had the lowest (11.15%).

**Figure 3:**
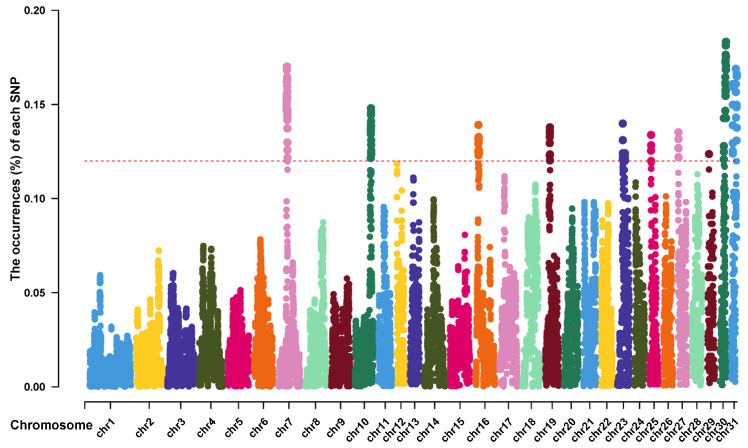
Manhattan plot of the occurrences (%) of each SNP within ROH regions in Thoroughbred population. Each colorful dot stands for an SNP. The horizontal red dotted line represents the cutoff level (top 1%).

Next, we examined the ROH islands in the Thoroughbred population to identify genomic regions that might have been subjected to selection pressure. We calculated the frequency of SNPs occurring in ROHs and selected the top 1% as an indicator of the ROH islands. The frequency of SNP occurrence within the ROH regions was plotted against the locations of the SNPs along the chromosome for each individual using the Manhattan plot. A total of 24 ROH islands containing 72 candidate genes were identified on ECA7, 10, 16, 19, 23, 25, 27, 29, 30, and 31 (Figure 3). The longest ROH island was identified on ECA16 with 3325 contiguous SNPs, whereas the shortest was observed on ECA31. ECA30 had the largest number of ROH islands (six ROH islands, including five candidate genes). 

Most identified ROH islands in Thoroughbreds contained candidate genes. However, six ROH islands on ECA25, 29, 30, and 31 did not contain any annotated protein-coding genes. Enrichment analyses for GO and KEGG on all identified candidate genes were conducted. Nine significant GO biological process terms and three significant KEGG pathways are listed in Supplementary Table S3. The most significantly enriched GO terms were neurological signaling, neuronal development, positive regulation of heart rate and contraction, and metabolic processes. KEGG pathways were significantly enriched in cholinergic synapses, retrograde endocannabinoid signaling, and insulin secretion. We found that the candidate genes were involved in neurotransmission (*CHRNA6*, *PRKN*, and *GRM1*), muscle development (*ADAMTS15* and *QKI*), positive regulation of heart rate and contraction (*HEY2* and *TRDN*), regulation of insulin secretion (*CACNA1S*, *KCNMB2*, and *KCNMB3*), and spermatogenesis (*JAM3*, *PACRG*, and *SPATA6L*). 

## 4. Discussion

### 4.1. Distribution and Patterns of ROH in 16 Horse Populations

In the diploid genome, ROHs are the contiguous regions in which all SNPs at any position are homozygous in an individual [13]. In our study, we examined the length patterns of ROH in 16 diverse horse populations. In general, short ROHs (1 Mb) were the most prevalent, followed by medium (1–5 Mb) and medium-long ROHs (5–10 Mb), with only a dozen ultra-long ROHs (>10 Mb) detected. The ROH lengths may approximate the period during which inbreeding occurs. For instance, short ROHs indicate a history of ancestral inbreeding, whereas long ROHs usually result from recent inbreeding events. We found that the average length of the short ROHs was much longer in horse breeds (such as Friesian, Thoroughbred, and Arabian) that had been subjected to strong artificial selection than in native horse breeds (such as Mongolian, Debao, and Yakutian). 

In conjunction with the number and average length of ROHs based on the length categories, the results suggested that ancient and recent inbreeding events may have varying degrees of influence on various horse breeds. However, very recent instances of inbreeding were uncommon, particularly among indigenous horse breeds. It is worth noting that inbreeding events are not the only factor affecting ROH length. Owing to dynamic randomness and recombination during gamete formation, the generation and evolution of ROHs are random events to a certain extent [26]. In addition, reduced population size and bottlenecks may alter the properties of short ROH (<4 Mb) [27]. 

### 4.2. ROH-Based Genomic Inbreeding Coefficients

Traditionally, the inbreeding coefficient has been calculated primarily using data obtained from pedigrees. However, the horse pedigree records often contain errors that may have occurred long ago and could not be tracked. On the other hand, some native horse breeds did not even have pedigree records. Recently, calculating inbreeding coefficients using the genome-wide SNP data of livestock is now achievable thanks to the advent of high-density SNP genotyping technology (such as SNP chips and whole-genome sequencing) [19]. SNP data are more advantageous than pedigree data for evaluating the impact of inbreeding [28]. Moreover, SNP-based calculations of the inbreeding coefficients demonstrated authentic relationships between individuals [29]. 

In our study, we used the whole-genome sequencing method to estimate unbiased genome-wide inbreeding coefficients. We found that horse breeds that required breed registrations and had studbooks had high overall inbreeding coefficients (high *F*_ROH all_). For example, due to the limited number of Thoroughbred founders, their effective population size is modest. In contrast, indigenous horse breeds showed relatively low degrees of inbreeding (low *F*_ROH all_). We further calculated the *F*_ROH_ using different lengths of ROH as follows: *F*_ROH < 1 Mb_, *F*_ROH 1–5 Mb_, *F*_ROH 5–10 Mb_, and *F*_ROH > 10 Mb_, which reflect, respectively, ancestral inbreeding events that happened 50 generations, 10–50 generations, 5–10 generations, and 5 generations ago [30]. All 16 horse breeds have historical inbreeding events dating back to 50 generations. Only three horse breeds (Thoroughbred, Standardbred, and Franches-Montagnes) had *F*_ROH > 10 Mb_, indicating that inbreeding events occurred within five generations. Overall, the ROH-based genomic inbreeding coefficient can be useful for estimating the inbreeding levels of individual horses lacking pedigree information. It could also provide useful indicators for monitoring increases in inbreeding, preserving horse breeds, and minimizing the adverse impacts of inbreeding on horse populations.

### 4.3. Candidate Genes in ROH Islands in Thoroughbreds Are Associated with Artificial Selection Traits

ROH can be employed to define genomic regions subject to selection pressure and to characterize the occurrence of selective sweeps. Using the Thoroughbred population as a case study, we evaluated the candidate genes within the ROH islands. In contrast to other domesticated animals, horses are valued for their temperament. Important for the breeding, selection, and training of horses, temperament is defined as an innate neurological characteristic. Due to the fact that the Thoroughbred horse has traditionally been characterized as a “hot blood” breed and their temperament has been described as extremely prone to nervousness [31], several candidate genes discovered by our analysis have been reported to play crucial roles in neurotransmission. For example, *CHRNA6* encodes an α subunit of the neuronal nicotinic acetylcholine receptor that regulates dopaminergic neurotransmission. In humans, mutations in this gene most likely result in neuropsychiatric disorders (autism, depression, bipolar disorder, and schizophrenia), neurodegenerative diseases (Parkinson’s and Alzheimer’s disease), and lung cancer [32,33]. *PRKN* encodes Parkin, a component of the E3 ubiquitin ligase complex, and mutations in this gene have been implicated in Parkinson’s disease [34] and Autism spectrum disorder [35]. In addition, Prkn-deficient mice exhibit autistic-like behavior and defective synaptogenesis [36]. The metabotropic glutamate receptor, which is encoded by the *GRM1* gene, is involved in learning, synaptic activity, and neuroprotection. It is also associated with inherited cerebellar ataxia [37]. 

Thoroughbreds are considered to have great athletic ability because their maximum oxygen uptake (VO_2max_) is nearly double that of elite human athletes [38,39]. Equine scientists and breeders believe that Thoroughbreds must strengthen their cardiorespiratory capacity and muscle adaptation to obtain such high athletic ability. Consequently, it is possible that the cardiovascular and muscular systems of Thoroughbreds have been subjected to intense artificial selection. Several candidate genes associated with cardiac development have been identified. For example, *HEY2* encodes a member of the basic Helix-Loop-Helix (bHLH) subfamily. It has been suggested that *HEY2* controls heart growth by limiting cardiomyocyte proliferation [40] and is considered a crucial regulator of human cardiac development [41]. Triadin, one of the major cardiac sarcoplasmic reticulum proteins encoded by *TRDN*, stimulates muscle contraction via calcium-induced calcium release [42]. Humans and mice exhibited aberrant heart rates due to the loss of function of *TRDN* [43]. In addition, we identified candidate genes associated with muscle development, such as myoblast fusion (*ADAMTS15*) [44] and vascular smooth cell differentiation (*QKI*) [45]. 

Insulin is secreted by pancreatic β-cells to increase glucose consumption by promoting glucose uptake, glycogen synthesis, and adipogenesis in muscle and adipose tissue [46]. Insulin is essential for maintaining glucose homeostasis in the body. Studies have demonstrated that insulin secretion is a complex process in which sodium, potassium, and calcium channels in the membrane of pancreatic β-cells play crucial roles [47,48]. Thoroughbred horses are insulin-sensitive [49], and insulin stimulates muscle and protein synthesis [50]. Several candidate genes were significantly associated with insulin secretion regulation in our study. For instance, *KCNMB2* and *KCNMB3* are two potassium calcium-activated channel genes inherited in the linkage region, and *CACNA1S* encodes the voltage-gated calcium channel subunit α Ca_V_1.1, which may be jointly involved in regulating insulin secretion in Thoroughbreds.

Since the vast majority of the sequenced Thoroughbreds we used were males, we also identified candidate genes involved in spermatogenesis (*JAM3*, *PACRG*, and *SPATA6L*). The adhesion of germ and Sertoli cells regulates the dynamic process of spermatogenesis. Junctional adhesion molecule-C (JAM-C, encoded by *JAM3*) is expressed by germ cells and localizes to the junctions between germ and Sertoli cells. JAM-C participates in the formation of acrosomes and germ cell polarity [51]. The development of the flagellum is a crucial step in spermiogenesis because it enables sperm to reach the egg for fertilization. A MEIG1/PACRG complex in the manchette transports cargo to the centrioles, which are used to construct sperm tails [52]. Although *SPATA6L* (encoding spermatogenesis-associated 6-like protein) is predicted to be located in sperm connecting pieces and to be involved in spermatogenesis, its molecular function remains unknown. An important paralog of *SPATA6L* is *SPATA6*, which is necessary for the correct assembly of the sperm connecting component and head-tail junction [53]. In the artificial selection of Thoroughbreds for breeding, athletic performance and superior pedigree lines take precedence over reproductive fitness. Therefore, almost no selection pressure was exerted on fertility traits [54,55]. Typically, the conception rate of Thoroughbreds is lower than that of other livestock breeds, at about 60% per conception cycle [56]. All registered foals in the Thoroughbred horse industry must be born naturally, and artificial reproduction techniques are prohibited. In addition, breeding seasons in the Northern and Southern Hemispheres are strictly regulated by the industry. We hypothesized that the relaxation of reproductive traits could result in the accumulation of deleterious mutations that could diminish the reproductive ability of Thoroughbred stallions. These candidate genes associated with spermatogenesis may serve as targets for the future selection of Thoroughbreds in an effort to improve stallion fertility.

## 5. Conclusions

The present study examined the distribution of ROH and estimated inbreeding coefficients based on ROH in 16 diverse horse breeds using whole-genome sequencing data from 97 horses. Our data suggest that ancient and recent inbreeding may affect horse breeds differently, but recent inbreeding is uncommon, particularly among indigenous horse breeds. The ROH-based genomic inbreeding coefficient is useful for estimating horse inbreeding levels in horses without pedigree data and for monitoring inbreeding increments in the horse population. Moreover, we identified 24 ROH islands containing 72 candidate genes associated with artificial selection traits in Thoroughbreds. These candidate genes are associated with neurotransmission, muscle development, positive regulation of heart rate and contraction, regulation of insulin secretion, and spermatogenesis. These findings provide insight into the characteristics of horse breeds and future breeding strategies.

## Figures and Tables

**Table 1:** Summary statistics of the runs of homozygosity (ROH) based on length classes.

							Classification of ROH by Length			
Horse Population	No. of Samples	Total No. ^a^	Mean No. ^b^	Total Length (Mb) ^c^	Total Mean Length (Mb) ^d^	Max. Length (Mb) ^e^	<1 Mb	1–5 Mb	5–10 Mb	>10 Mb
Friesian	5	3183	637	3178.47	635.69	7.82	2116	1060	7	0
Thoroughbred	22	12,490	568	13,526.86	614.86	13.89	8233	4130	117	10
Arabian	5	3104	621	3013.17	602.63	7.34	2119	970	15	0
Shetland pony	3	1435	478	1545.37	515.12	8.18	918	504	13	0
Andalusian	4	2102	526	2047.94	511.99	8.47	1428	667	7	0
Akhal-Teke	5	2112	422	1997.03	399.41	8.37	1508	599	5	0
Standardbred	4	1549	387	1550.21	387.55	11.38	1094	437	17	1
Hanoverian	4	1282	321	1207.67	301.92	7.32	918	360	4	0
American Quarter Horse	7	2173	310	1997.14	285.31	8.99	1587	575	11	0
Franches-Montagnes	6	1476	246	1649.65	274.94	10.56	939	520	16	1
Criollo	2	491	246	403.68	201.84	4.12	390	101	0	0
Jeju	2	501	251	369.51	184.76	4.13	417	84	0	0
Przewalskii	10	1802	180	1591.60	159.16	8.74	1378	423	1	0
Mongolian	5	993	199	785.24	157.05	4.70	809	184	0	0
Debao	5	836	167	593.08	118.62	5.06	712	123	1	0
Yakutian	7	711	102	459.81	65.69	2.91	639	72	0	0

^a^ Total No.: The overall amount of ROH found in a horse population. ^b^ Mean No.: the average number of total ROH per horse breed. ^c^ Total Length: sum of all ROH lengths obtained within a horse population. ^d^ Total Mean Length: the average of the total length of ROH in each population. ^e^ Max. Length: maximum length of ROH segment detected in a horse population.

**Table 2:** Mean genomic inbreeding coefficients (*F*_ROH_) for ROH of different length categories in horse populations.

			ROH Length Category (Mb)					
Horse Population	Horse Population	No. of Samples	*F*_ROH_ (<1 Mb)	*F*_ROH_ (1–5 Mb)	*F*_ROH_ (5–10 Mb)	*F*_ROH_ (>10 Mb)	*F*_ROH all_	SD
FS	Friesian	5	1.16 × 10^−1^	1.59 × 10^−1^	3.81 × 10^−3^	0	2.79 × 10^−1^	3.11 × 10^−4^
AB	Arabian	5	1.15 × 10^−1^	1.41 × 10^−1^	7.50 × 10^−3^	0	2.64 × 10^−1^	3.12 × 10^−4^
TB	Thoroughbred	23	9.73 × 10^−2^	1.44 × 10^−1^	1.42 × 10^−2^	2.27 × 10^−3^	2.58 × 10^−1^	4.25 × 10^−4^
AL	Andalusian	4	9.69 × 10^−2^	1.23 × 10^−1^	4.87 × 10^−3^	0	2.24 × 10^−1^	3.13 × 10^−4^
ST	Shetland pony	3	8.26 × 10^−2^	1.31 × 10^−1^	1.19 × 10^−2^	0	2.26 × 10^−1^	3.93 × 10^−4^
AT	Akhal-Teke	5	8.14 × 10^−2^	9.06 × 10^−2^	3.06 × 10^−3^	0	1.75 × 10^−1^	3.17 × 10^−4^
STD	Standardbred	4	7.33 × 10^−2^	8.39 × 10^−2^	1.15 × 10^−2^	1.25 × 10^−3^	1.70 × 10^−1^	3.93 × 10^−4^
HAN	Hanoverian	4	6.00 × 10^−2^	6.95 × 10^−2^	2.79 × 10^−3^	0	1.32 × 10^−1^	3.25 × 10^−4^
QT	American Quarter Horse	7	5.94 × 10^−2^	6.15 × 10^−2^	4.22 × 10^−3^	0	1.25 × 10^−1^	3.26 × 10^−4^
JEJU	Jeju	2	5.41 × 10^−2^	2.69 × 10^−2^	0	0	8.10 × 10^−2^	ND
CR	Criollo	2	5.19 × 10^−2^	3.66 × 10^−2^	0	0	8.85 × 10^−2^	ND
MON	Franches-Montagnes	5	4.96 × 10^−2^	8.53 × 10^−2^	8.80 × 10^−3^	9.26 × 10^−4^	1.45 × 10^−1^	4.43 × 10^−4^
MG	Mongolian	5	4.26 × 10^−2^	2.63 × 10^−2^	0	0	6.89 × 10^−2^	2.21 × 10^−4^
DB	Debao	5	3.54 × 10^−2^	1.62 × 10^−2^	4.44 × 10^−4^	0	5.20 × 10^−2^	1.90 × 10^−4^
PRZ	Przewalskii	10	3.50 × 10^−2^	3.44 × 10^−2^	3.83 × 10^−4^	0	6.98 × 10^−2^	3.10 × 10^−4^
YAK	Yakutian	7	2.26 × 10^−2^	6.20 × 10^−3^	0	0	2.88 × 10^−2^	1.33 × 10^−4^

Note: *F*_ROH_ was calculated using this formula: *F*_ROH_ = *L*_ROH_/*L*_AUTO._ The total length of ROH on autosomes is denoted by *L*_ROH_. *L*_AUTO_ is the total autosomal length (2280.94 Mb). ND: not detected. SD: standard deviation (SD is only calculated for population sample sizes greater than 3).

## Data Availability

The whole genome data used in this manuscript are available in the GenBank database under BioProject accession PRJNA416233, PRJEB10098, PRJEB10854, PRJNA168142, PRJNA205517, PRJNA230019, PRJNA233529, PRJNA288817 and PRJNA291776.

## Supplementary Materials

The following supporting information can be downloaded at: https://www.mdpi.com/article/10.3390/genes14061211/s1, Table S1: Mapping results of clean reads against horse reference genome; Table S2: The statistics of ROH on each chromosome in the Thoroughbred population; Table S3: The top functional categories enriched for candidate genes located in ROH islands in Thoroughbreds.
