# Supplementary material for: Genome-Wide Assessment of Runs of Homozygosity by Whole-Genome Sequencing in Diverse Horse Breeds Worldwide
Source: Genes (Basel). 2023 Jun 1;14(6):1211. doi: 10.3390/genes14061211 (PMC10298080; doi:10.3390/genes14061211)
Supplement: Supplementary file 1 [file genes-14-01211-s001.zip › genes-2392487-supplementary.pdf]

Genome-wide assessment of runs of homozygosity by whole-genome sequencing in diverse horse breeds worldwide

<u>Supplementary files</u>

Table S1. Mapping results of clean reads against horse reference genome.

Sample	Clean reads	Mapped reads	Mapping rate (%)	Average depth (X)	Coverage at least 1X (%)	Coverage at least 4X (%)
AB_17A	471333056	469131719	0.9953	24.98	0.9835	0.9485
AB_2	1465449498	1442850879	0.9846	70.42	0.991	0.9758
AB_24A	704078740	700436947	0.9948	36.5	0.9886	0.9631
AB_24B	483069738	480439075	0.9946	25.52	0.9843	0.9507
AB_MET	371662830	359474445	0.9672	14.67	0.9779	0.9255
AL_12A	1272099926	1265483272	0.9948	64.17	0.9913	0.9753
AL_12B	596912856	594116950	0.9953	31.47	0.9857	0.9555
AL_12C	522077762	519352038	0.9948	27.7	0.9858	0.9531
AL_12D	501115756	497575978	0.9929	26.18	0.9846	0.951
AT_19	477872184	474976981	0.9939	24.89	0.9847	0.9508
AT_23A	555574922	552432275	0.9943	30	0.9869	0.9564
AT_23C	651144578	646733923	0.9932	35.14	0.9889	0.9618
AT_3	1307683510	1299154576	0.9935	65.28	0.9915	0.9769
AT_5	449808036	447410324	0.9947	23.73	0.9841	0.9496
CR_10	455050378	453076568	0.9957	24.14	0.9839	0.9495
CR_4	1501677796	1493422745	0.9945	76.46	0.9931	0.9802
DB_14A	1346581062	1334857939	0.9913	69.07	0.9916	0.9753
DB_14B	442554626	440585471	0.9956	23.7	0.9842	0.9493
DB_14C	454130138	452339864	0.9961	24.21	0.9838	0.9482
DB_14D	634080784	631454414	0.9959	33.15	0.9887	0.9604
DB_14E	504937958	502667784	0.9955	26.5	0.9853	0.9522
FS_11A	1257054024	1249607080	0.9941	64.57	0.989	0.9713
FS_11B	492508732	490484758	0.9959	26.12	0.9841	0.9517
FS_11C	448653154	446801396	0.9959	23.8	0.9828	0.9488
FS_11D	494933218	493017897	0.9961	26.19	0.9843	0.9521
FS_11E	471181678	468792849	0.9949	25.72	0.9825	0.948
HAN1	695723316	669180169	0.9618	26.6	0.9887	0.9533
HAN2	307104000	291931975	0.9506	12.08	0.973	0.9109
HAN3	295239106	290625783	0.9844	12.2	0.9734	0.9048
HAN4	279654242	274951397	0.9832	11.61	0.9724	0.8973
JEJU1	400304134	388880872	0.9715	13.98	0.9775	0.912
JEJU2	403284330	394627782	0.9785	13.67	0.9773	0.905
MG_16A	1420917578	1399822152	0.9852	67.11	0.9916	0.975
MG_16B	488242312	484891796	0.9931	25.43	0.9849	0.9501

MG_16C	495659876	493130560	0.9949	26.16	0.9847	0.9493
MG_D2628	670705898	666239953	0.9933	26.14	0.9843	0.9413
MG_D2629	893708808	887577234	0.9931	33.73	0.9888	0.9558
MON_FM0450	398669810	397050697	0.9959	16.45	0.9718	0.9166
MON_FM1030	435248512	432172536	0.9929	17.35	0.973	0.9241
MON_FM1190	412950454	410573136	0.9942	16.52	0.9723	0.9221
MON_FM1798	551612288	547671135	0.9929	22	0.9775	0.9352
MON_FM1951	491849372	488580384	0.9934	19.48	0.976	0.9297
PRZ_D2630	575973260	571359508	0.992	22.16	0.9813	0.9311
PRZ_D2631	735775306	731227103	0.9938	28.3	0.9855	0.9463
PRZ_SB159	421145912	416580331	0.9892	15.1	0.9702	0.9124
PRZ_SB274	262067206	259008746	0.9883	10.08	0.9497	0.8275
PRZ_SB281	407830422	399720934	0.9801	14.23	0.9696	0.9061
PRZ_SB285	309606552	305921145	0.9881	11.71	0.9573	0.8675
PRZ_SB339	335191688	331522803	0.9891	12.4	0.9601	0.8804
PRZ_SB524	496950092	492304055	0.9907	17.34	0.9748	0.9296
PRZ_SB615	306239644	302146935	0.9866	11.15	0.9579	0.8618
PRZ_SB966	374263918	370707173	0.9905	13.81	0.9684	0.9096
QT_22A	1414382142	1403659890	0.9924	70.82	0.9935	0.9826
QT_22B	445582284	442025912	0.992	24.11	0.9853	0.9506
QT_22C	510163930	507754657	0.9953	27.73	0.9861	0.9546
QT_A1543	282160770	280598076	0.9945	11.68	0.9634	0.8802
QT_A2085	279585894	278001549	0.9943	11.73	0.9615	0.867
QT_A5659	342193142	339978914	0.9935	13.84	0.969	0.9035
QT_A5964	342108290	339918219	0.9936	13.8	0.9652	0.9002
ST_13A	1364181834	1354624556	0.993	69.27	0.9914	0.9755
ST_SPH020	629763348	625849185	0.9938	24.85	0.9872	0.951
ST_SPH041	572600368	568488749	0.9928	22.68	0.9857	0.9476
STD_M1009	225581310	224189163	0.9938	9.69	0.9628	0.8421
STD_M5256	260763222	259073713	0.9935	10.8	0.9607	0.8658
STD_M977	297562442	295915553	0.9945	12.01	0.9652	0.8983
STD_ST22	874232206	872044754	0.9975	32.12	0.9895	0.9593
TB_5	545231814	542254795	0.9945	27.66	0.9865	0.957
TB_6	528018910	524231041	0.9928	26.85	0.9863	0.9547
TB_7	1299193532	1291659229	0.9942	66.81	0.9922	0.9772
TB_8	558480678	555461210	0.9946	28.11	0.9878	0.9589
TB_9	542264920	539395586	0.9947	27.3	0.9867	0.9574
TB_TB03	837984126	836047281	0.9977	30.98	0.9896	0.9586
TB_TB10	867060418	865158724	0.9978	31.71	0.9888	0.9585
TB_thb1	436349272	425060441	0.9741	13.07	0.9782	0.9021
TB_thb10	388213896	384599221	0.9907	15.37	0.9793	0.9228
TB_thb11	470077452	455929397	0.9699	13.81	0.9805	0.9156
TB_thb12	479312482	469927384	0.9804	16.53	0.9819	0.9285
TB_thb13	464112948	458567526	0.9881	17.74	0.9812	0.9318

TB_thb14	457444368	454469990	0.9935	18.38	0.9819	0.9332
TB_thb2	431590084	415548384	0.9628	12.86	0.9783	0.9034
TB_thb3	436349272	425060441	0.9741	13.07	0.9782	0.9021
TB_thb4	422333114	409962038	0.9707	14.56	0.9794	0.9188
TB_thb5	419238334	408399681	0.9741	13.83	0.9784	0.9135
TB_thb6	466451738	454308682	0.974	14.45	0.9798	0.9167
TB_thb7	483638634	473170070	0.9784	14.25	0.9799	0.9135
TB_thb8	390136878	377909067	0.9687	13.14	0.9761	0.902
TB_thb9	399429568	388950915	0.9738	12.45	0.9768	0.9041
TB_TWI	742581666	739877687	0.9964	28.35	0.9868	0.9479
YAK1	553205004	543780184	0.983	18.93	0.9739	0.9277
YAK2	897135906	886153834	0.9878	29.71	0.9862	0.9555
YAK3	548175044	536315398	0.9784	18.47	0.9748	0.9324
YAK4	467721960	453811212	0.9703	16.08	0.9689	0.9126
YAK5	552779254	540225170	0.9773	18.7	0.9737	0.9306
YAK6	600858092	591688805	0.9847	20.5	0.9763	0.9365
YAK7	1118670734	1101118366	0.9843	36.31	0.988	0.9611
YAK8	457535456	449313223	0.982	16.19	0.9706	0.9163
YAK9	466595932	455458059	0.9761	16.33	0.9717	0.9196

Table S2. The statistics of ROH on each chromosome in the Thoroughbred population.

Chr	Total No.of ROH	ROH total length (bp)	Length of each Chr (bp)	ROH Coverage (%)
Chr1	997	53087.12365	188260.577	28.20
Chr2	522	24050.38857	121350.024	19.82
Chr3	550	26526.95657	121351.753	21.86
Chr4	559	31820.77617	109462.549	29.07
Chr5	494	21701.48226	96759.418	22.43
Chr6	516	21236.28509	87230.776	24.34
Chr7	441	21237.26952	100787.686	21.07
Chr8	426	20135.38122	97563.019	20.64
Chr9	370	17663.77391	85793.548	20.59
Chr10	359	16762.71391	85155.674	19.68
Chr11	341	13635.25674	61676.917	22.11
Chr12	115	4124.997174	36992.759	11.15
Chr13	163	6170.784435	43784.481	14.09
Chr14	432	23580.80765	94600.235	24.93
Chr15	371	17871.08978	92851.403	19.25
Chr16	376	22113.27822	88962.352	24.86
Chr17	432	25531.71948	80722.43	31.63
Chr18	404	24606.48257	82641.348	29.78
Chr19	290	13276.84013	62681.739	21.18
Chr20	298	13838.44691	65343.332	21.18
Chr21	290	15074.83822	58984.458	25.56
Chr22	273	11266.40852	50928.189	22.12
Chr23	248	11995.27387	55556.184	21.59
Chr24	221	9146.348478	48288.683	18.94
Chr25	156	6086.842696	40282.968	15.11
Chr26	216	9018.652348	43147.642	20.90
Chr27	171	7170.719087	40254.69	17.81
Chr28	220	9033.619348	47348.498	19.08
Chr29	102	4163.748478	34776.12	11.97
Chr30	146	6808.866696	31395.959	21.69
Chr31	132	6188.802783	26001.039	23.80

Table S3. The top functional categories enriched for candidate genes located in ROH islands in Thoroughbreds.

**Category**	**Term description**	**Involved gene number**	***P* value (EASE scores)**
KEGG-pathway:ecb04725	Cholinergic synapse	5	6.89E-04
GO:0007286	spermatid development	3	1.61E-02
KEGG-pathway:ecb04723	Retrograde endocannabinoid signaling	4	1.68E-02
GO:0005513	detection of calcium ion	2	2.26E-02
GO:0098712	L-glutamate import across plasma membrane	2	2.64E-02
GO:0010460	positive regulation of heart rate	2	3.01E-02
GO:0042417	dopamine metabolic process	2	3.38E-02
KEGG-pathway:ecb04911	Insulin secretion	3	3.83E-02
GO:0046039	GTP metabolic process	2	4.11E-02
GO:0043524	negative regulation of neuron apoptotic process	3	4.25E-02
GO:0019221	cytokine-mediated signaling pathway	3	4.61E-02
GO:0060047	heart contraction	2	4.84E-02
